# A Rapid p72‐Targeting Colloidal Gold Immunochromatographic Strip for African Swine Fever Virus Detection

**DOI:** 10.1155/tbed/3198773

**Published:** 2026-06-05

**Authors:** Wan Wang, Weldu Tesfagaber, Wenzhuang Zhu, Li Yin, Fan Liu, Runyi Yao, Shangcong Wu, Fang Li, Encheng Sun, Zhenjiang Zhang, Zhigao Bu, Geng Meng, Yuanmao Zhu, Dongming Zhao

**Affiliations:** ^1^ State Key Laboratory of Animal Disease Control and Prevention, Professional Laboratory for African Swine Fever (Harbin), National High Containment Facilities for Animal Diseases Control and Prevention, Harbin Veterinary Research Institute, Chinese Academy of Agricultural Sciences, Harbin, 150069, China, caas.cn; ^2^ College of Veterinary Medicine, China Agricultural University, Beijing, 100083, China, cau.edu.cn

**Keywords:** African Swine Fever Virus, colloidal gold immunochromatography strip, detection, p72 protein

## Abstract

African swine fever (ASF) is a lethal, hemorrhagic, and highly contagious infectious disease caused by the African swine fever virus (ASFV). At present, ASFV has evolved into multiple variant strains with different levels of virulence in China, complicating the epidemiological landscape. In the absence of effective commercial vaccines, the development of rapid and accurate diagnostic methods has become a critical priority for epidemic prevention and control. In this study, monoclonal antibodies (mAbs) were generated by immunizing mice with inactivated ASFV. Nine newly identified mAbs targeting the p72 protein were screened to establish a colloidal gold immunochromatographic strip for ASFV detection combined with 11 previously characterized anti‐p72 mAbs developed in our laboratory. Cross‐pairing analysis was subsequently performed to identify the optimal antibody combination. Based on this evaluation, ASFV‐3 and P72‐6 were selected as the gold‐conjugated antibody and capture antibody, respectively. The assay demonstrated the capacity to effectively detect diverse ASFV genotypes, with a limit of detection (LOD) of 10^2.50^ TCID_50_ per reaction against genotype Ⅰ and Ⅱ ASFVs. No cross‐reactivity was observed with five common swine pathogens including PCV, PRRSV, PRV, PEDV, and TGEV. The concordance rate with the results from commercially available test strips was 93.33%. In conclusion, a rapid, sensitive, and highly specific colloidal gold immunochromatographic strip targeting the ASFV p72 protein was successfully developed. This method is simple to operate, does not require specialized equipment, and is suitable for on‐site detection, providing a valuable tool for field surveillance and early diagnosis of ASF.

## 1. Introduction

African swine fever (ASF), caused by the African swine fever virus (ASFV), is a highly lethal, acute hemorrhagic disease affecting domestic pigs and wild boars [1, 2]. It currently represents one of the most significant threats to the global swine industry [3, 4]. ASF is now geographically widespread; according to the World Organization for Animal Health (WOAH), outbreaks have been reported in 83 countries, with 13 countries reporting their first cases since 2022 [5]. From January 2022 to November 2025, over 41 thousand outbreaks were documented, resulting in the loss of ~2.31 million pigs [5]. China reported its first ASF case in August 2018 [6], identified as a highly virulent genotype Ⅱ strain [7]. Since then, the epidemiological situation has grown increasingly complex with the subsequent emergence of moderately virulent genotype Ⅱ strains [8], low‐virulence genotype I strains [9], and highly virulent genotype Ⅰ/Ⅱ recombinant strains [10], posing substantial new challenges for national prevention and control programs.

ASFV, the sole member of the *Asfivirus* genus within the Asfarviridae family, possesses a large, double‐stranded linear DNA genome (170–194 kb) encoding 151–167 open reading frames [1, 11, 12]. Mature virions are spherical, ~260–300 nm in diameter, and exhibit a complex multilayered architecture comprising an outer envelope, capsid, inner membrane, nucleoprotein core shell, and genomic core [3, 13]. The p72 protein, encoded by the B646L gene, is the major capsid protein. It forms trimers on the viral surface, constituting roughly 33% of the virion’s total mass [14]. The p72 protein is highly conserved across strains and exhibits strong immunogenicity, making it a principal target for diagnostic assays and vaccine development efforts [15, 16]. Due to the virus’s intricate structure, extensive genome, and insufficient understanding of its immune evasion mechanisms and correlates of protection [17], no safe and effective commercial vaccines or antiviral therapies are currently available [18, 19]. Consequently, disease management relies heavily on stringent biosecurity measures and precise outbreak containment strategies.

Effective ASF control is fundamentally dependent on reliable diagnostic capabilities, particularly the availability of rapid and accurate on‐site pathogen detection for timely epidemiological responses. While nucleic acid amplification techniques such as quantitative PCR (qPCR) offer high sensitivity, they are prone to potential false positives, require sophisticated instrumentation and skilled personnel, and are not readily adaptable for field or grassroots application. Therefore, there is a pressing need to develop rapid, accurate, user‐friendly, and instrument‐free diagnostic technologies suitable for field settings. Colloidal gold immunochromatography technology presents an ideal solution, offering advantages of speed, simplicity, low cost, and minimal operational requirements [20]. Leveraging this platform, this study successfully developed a colloidal gold‐based lateral flow strip for the rapid presumptive detection of ASFV, aiming to provide a more accessible and effective on‐site diagnostic tool for epidemic surveillance and control.

## 2. Materials and Methods

### 2.1. Cells, Viruses, and Biological Materials

Mouse myeloma cells (SP2/0), wild boar kidney cells (BK2258), primary porcine alveolar macrophages (PAM), and primary porcine bone marrow cells (PBM) were cultivated and maintained by the Harbin Veterinary Research Institute (HVRI), Chinese Academy of Agricultural Sciences (CAAS).

Viral strains, including ASFV HLJ/18‐7GD (seven gene‐deleted, genotype Ⅱ) [21], ASFV HLJ/18 (genotype Ⅱ) [7], ASFV HLJ/HRB1/20 (genotype Ⅱ) [8], ASFV SD/DY‐Ⅰ/21 (genotype Ⅰ) [9], ASFV JS/LG/21 (genotype Ⅰ/Ⅱ recombinant) [10], ASFV SD (the subculture‐adapted strain of ASFV SD/DY‐Ⅰ/21 in BK2258 cells), PCV‐2d, PRRSV HuN4, PRV HeN1, PEDV LN‐NY, and TGEV JMS, were isolated, identified, and preserved at the HVRI, CAAS. All experiments involving live ASFV were conducted in the Biosafety Level 3 (P3) Laboratory at the HVRI and have been approved by the Biosafety Committee of the State Key Laboratory of Animal Disease Prevention and Control.

The p72 protein with a natural trimeric structure was prepared and provided by China Agricultural University. 11 mAbs against p72 were previously prepared and preserved in our laboratory [15]. In addition, p30 protein was obtained by transfecting Expi293F suspension cells with pcDNA3.4‐p30 plasmid, which contains the CP204L gene coding sequence from the ASFV HLJ/18 strain (GenBank: MK333180.1), followed by induction of exogenous protein expression and purification using a His‐tag purification system.

### 2.2. ASFV Cultivation, Inactivation, and Purification

The ASFV HLJ/18‐7GD strain was inoculated into PBM cells at a multiplicity of infection (MOI) of 0.1 and cultured at 37°C with 5% CO_2_ until cytopathic effect (CPE) reached ~80%. The viral suspension was harvested, subjected to two freeze–thaw cycles at −70°C, and clarified by centrifugation (4°C, 5000 rpm, and 20 min). The supernatant containing crude virus was inactivated using β‐propiolactone at 4°C for 72 h. The inactivated virus was preliminarily concentrated via iodixanol density gradient centrifugation, further purified by continuous sucrose density gradient centrifugation, and finally buffer‐exchanged and resuspended in PBS for storage at −70°C.

### 2.3. Preparation and Purification of mAbs

Six to eight‐week‐old SPF female BALB/c mice were immunized with inactivated ASFV emulsified in Freund’s adjuvant. The primary immunization used complete Freund’s adjuvant, while booster immunizations at 2‐week intervals used incomplete Freund’s adjuvant (100 μg antigen per dose). Serum antibody titers were evaluated 10–12 days postthird immunization by indirect ELISA (iELISA). The mouse exhibiting the highest titer received a final subcutaneous boost (100 μg inactivated virus). Splenocytes from this mouse were fused with SP2/0 myeloma cells. Hybridoma supernatants were screened by indirect immunofluorescence assay (IFA) using ASFV SD strain‐infected BK2258 cells as antigen. Positive clones were expanded, and mAbs were produced in mouse ascites, followed by purification via Protein G affinity chromatography.

### 2.4. IFA

BK2258 monolayers in 96‐well plates were infected with ASFV SD strain (MOI = 0.001) [22]. After 48 h, cells were washed with PBS, fixed with 4% paraformaldehyde (30 min, RT), and permeabilized with 0.25% Triton X‐100 (15 min, RT). After blocking, cells were incubated with hybridoma supernatants or control sera (1:200 dilution, 2 h, 37°C), followed by FITC‐conjugated goat antimouse IgG (1:100 dilution, 45 min, 37°C). Fluorescence was visualized using a fluorescence microscope.

### 2.5. Western Blot

Lysates from ASFV‐infected BK2258 cells were separated by 12.5% SDS‐PAGE and transferred to nitrocellulose (NC) membranes. Membranes were blocked with 5% skim milk, incubated with primary mAbs (1:200 dilution), washed, and probed with IRDye‐conjugated goat antimouse secondary antibody (1:10,000). Signal detection was performed using a near‐infrared imaging system.

### 2.6. IELISA

Recombinant proteins including A104R, B407L, B646L, CP312R, A137R, K145R, KP177R, K205R, CP204L, D117L, CP123L, and B602L were diluted to 4 μg/mL in carbonate buffer and coated onto ELISA plates overnight at 4°C. After 5 PBST washes, plates were blocked with 5% skimmed milk (prepared with PBS) 200 μL/well at 37°C for 2 h. After washing, 100 μL/well of mAbs diluted at 1:100 and 1:1000 were added and incubate at 37°C for 2 h. After frther washing, sheep anti‐mouse HRP enzyme label secondary antibody diluted at 1:10,000 was added, incubated at 37°C for 1 h, added TMB chromogenic solution (50 μL/well) after washing, and color was developed at 37°C in a dark area for 10 min. Finally, the reaction was terminated by adding 2 M sulfuric acid, and the OD_450_ value determined with a microplate reader within 5 min.

### 2.7. Preparation of Colloidal Gold Solution

A 40 nm colloidal gold solution was prepared by crystalline seed growth method, adapted from published protocols [23–25]. First, a 1% chloroauric acid solution, 0.2 mol/L sodium borohydride solution, and 0.04 mol/L trisodium citrate solution were prepared, respectively. Next, 200 mL of ultrapure water was placed in a 500 mL round‐bottom flask, followed by the addition of 2.8 mL of 1% chloroauric acid solution and 2 mL of 0.04 M trisodium citrate solution. After mixing, 4 mL of freshly prepared 0.2 M sodium borohydride solution was rapidly introduced under stirring. The reaction was allowed to proceed at room temperature for 30 min, yielding a colloidal gold seed suspension with an approximate particle size of 15 nm. Subsequently, 40 mL of ultrapure water and 1 mL of the above‐mentioned colloidal gold crystals were added to a 100 mL round‐bottomed flask under ice bath conditions, 30 μL trisodium citrate solution was added as a protective agent, 250 μL ascorbic acid solution was added as a reducing agent, and 200 μL chloroauric acid solution was slowly added after mixing, stirring continuously until the system gradually turned burgundy, the reaction continued for 30 min, and finally a colloidal gold solution with a particle size of about 40 nm was obtained. Dynamic light scattering (DLS, Malvern Zetasizer Nano ZS90) was used to determine the hydration particle size and polydispersity coefficient (PDI) of colloidal gold particles. At the same time, an appropriate amount of colloidal gold solution was added to the copper mesh, and the particle morphology was observed under transmission electron microscopy (TEM, Hitachi H‐7650) after drying.

The concentration of free antibodies in the solution before and after the conjugation reaction was determined by BCA protein quantification kit. The gold standard antibody solution was centrifuged at 4°C and 10,000 g for 20 min, and the supernatant was collected as unbound free antibody. The conjugation efficiency was calculated by the following formula: Conjugation efficiency (%) = (Total antibody concentration before conjugation − Free antibody concentration after conjugation)/Total antibody concentration × 100% before conjugation, with three independent replicates (*n* = 3) performed on all assays, and the results expressed as ± standard deviation of the mean.

### 2.8. Assembly of Antigen Test Strips

The pH of the 40 nm colloidal gold solution was adjusted to 7.5, followed by conjugation with ASFV‐3 mAb (20 μg/mL). The gold conjugate was purified by gradient centrifugation. After systematic optimization (as detailed in Table S1), the conjugate pad was treated with a solution containing BSA, PVP‐40, and sucrose, sprayed with the gold‐labeled antibody (8.0 μL/cm), and dried. On the NC membrane, the P72‐6 mAb (1.0 mg/mL) and goat antimouse IgG (2.0 mg/mL) were dispensed as the test (T) and control (C) lines, respectively, and dried at 37°C. The sample pad was pretreated with 0.02 M PBS (pH 7.4) containing 0.1% Triton X‐100% and 0.04% Proclin 300 (soaked for 30 s, drained, and dried at 60°C for 2 h). For clinical sample preparation, a sample diluent consisting of 0.02 M PBS (pH 7.4) containing 1% Triton X‐100% and 0.04% Proclin 300 was used. The strip assembly consisted of a sample pad, plasma separation membrane, conjugate pad, nitrocellulose membrane, and absorbent pad laminated sequentially onto a PVC backing. Strips were cut to 4 mm width, housed in cassettes, and sealed in aluminum foil pouches with desiccant.

### 2.9. Surface Plasmon Resonance (SPR) Affinity Assay

SPR analysis was performed using the Biacore 8K System (Cytiva). The monoclonal antibody to be tested was immobilized on the CM5 chip by amino conjugation at a fixed volume of 700–800 RU. Recombinant p72 protein was diluted with buffer (0.01 M PBS pH 7.4) to five concentration gradients (10, 5, 2.5, 1.25, and 0.625 μg/mL) and injected sequentially at a flow rate of 30 μL/min for 120 s binding time and 300 s dissociation time. The chip surface was regenerated using 10 mM glycine‐HCl (pH 2.0) after each injection. All data were deducted from the reference channel and blank injection background, and the binding rate constant (ka), dissociation rate constant (kd), and equilibrium dissociation constant (KD) were calculated using Biacore Insight Evaluation software for global fitting using a 1:1 Langmuir binding model.

### 2.10. Principle of the Test Strip Assay

The strip is designed for visual interpretation and is compatible with porcine oral/anal swabs, anticoagulated whole blood, and tissue homogenates. Prior to use, strips, samples, and sample diluent were equilibrated to room temperature. A total of 100 μL of processed sample (20 μL sample + 80 μL diluent) was applied to the sample well. Results were read after 10–15 min. In a positive sample, ASFV particles bind to the gold‐conjugated ASFV‐3 mAb, and the complex migrates chromatographically to be captured by the immobilized P72‐6 mAb at the T‐line, producing a purple‐red band. The remaining complex is captured by goat antimouse IgG at the C line. Interpretation: both T and C lines visible = positive; only C line visible = negative; C line not visible = invalid test.

### 2.11. Specificity of the Antigen Test Strips

To assess specificity, the strip was tested with high‐titer preparations of PCV‐2d (10^6.50^ TCID_50_/mL), PRRSV HuN4 (10^7.50^ TCID_50_/mL), PRV HeN1 (10^8.00^ TCID_50_/mL), PEDV LN‐NY (10^6.00^ TCID_50_/mL), TGEV JMS (10^6.70^ TCID_50_/mL), and ASFV HLJ/HRB1/20 (10^7.20^ TCID_50_/mL). Supernatants from uninfected PAM cells served as the negative control.

### 2.12. Sensitivity of the Antigen Test Strips

Serial tenfold dilutions of ASFV strains HLJ/HRB1/20 (10^7.20^ TCID_50_/mL), SD/DY‐Ⅰ/21 (10^7.12^ TCID_50_/mL), and JS/LG/21 (10^7.00^ TCID_50_/mL) were prepared in PBS and tested with the developed strips. All sensitivity tests were performed in three independent replicates. For parallel comparison, all samples were concurrently tested using the official TaqMan qPCR assay [26], with primers ASF‐05‐Zsak‐1466F/1528R and a FAM‐MGB probe. A sample was deemed qPCR‐positive if it showed a characteristic amplification curve with Ct ≤ 40.0; if Ct > 40.0, it was re‐tested and only considered positive upon re‐testing with Ct ≤ 40.0. Otherwise, it was recorded as negative. The detection limits of the CG‐ICS were then determined and compared with the qPCR results. In addition, a commercial test strip (Hangzhou Hengao Technology Co., Ltd.) was used to conduct simultaneous detection of the HLJ/HRB1/20 strain.

### 2.13. Reproducibility Test

To evaluate the intra‐ and interbatch reproducibility of the test strips, five clinical samples (P1–P5) confirmed ASFV‐positive by qPCR and five clinical samples (N1–N5) negative for ASFV were selected. Three different batches of test strips (batches 1, 2, and 3) were used, with each sample being tested three times per batch. The consistency of positive and negative results was used as the evaluation criterion for reproducibility.

### 2.14. Clinical Application of Antigen Test Strips

To validate the developed colloidal gold immunochromatographic test strip, we conducted a comparative analysis using samples from ASFV‐infected (HLJ/18 strain; *n* = 8) and healthy SPF (*n* = 1) pigs. From the infected group, oral and anal swabs were collected on 3, 5, 7, and 9 day postinfection (dpi). Tissue samples (spleen, lung, tonsil, submandibular lymph node, intestinal lymph node, and abdominal lymph node) were collected at the time of death from four of these pigs. Equivalent tissue samples were collected from the SPF‐control pig. In total, we tested 30 oral swabs, 30 anal swabs, 24 tissue homogenates from infected pigs, and 6 tissue homogenates from the SPF pig. All results were compared against both qPCR and a commercially available test kit (Hangzhou Hengao Technology Co., Ltd.).

## 3. Results

### 3.1. Animal Immunization and Antigen Preparation

**Figure 1:**
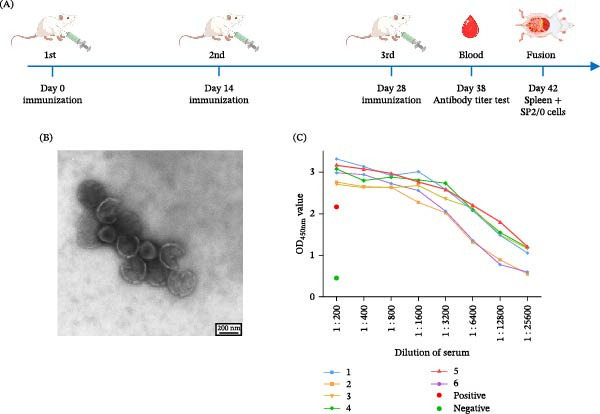
Preparation of the whole virus immunogen of ASFV and evaluation of its immunogenicity in mice. (A) Schematic overview of the immunization protocol. (B) Visualization of purified viral particles by electron microscopy. (C) Measurement of antigen‐specific antibody response. Sera obtained on day 38 were analyzed for antibodies against the major capsid protein p72 using an iELISA. Data are from a single experiment for preliminary assessment of postimmunization antibody titers.

Purification of ASFV via sucrose density gradient centrifugation yielded a distinct white band at the 30%–55% interface. TEM of this fraction confirmed the presence of intact and some disrupted virions (Figure 1B). Following the immunization schedule (Figure 1A), serum from all mice demonstrated strong anti‐p72 antibody responses by iELISA (Figure 1C), among which the highest titer number 5 mouse was selected for splenocyte fusion.

### 3.2. Generation and Characterization of mAbs

**Figure 2:**
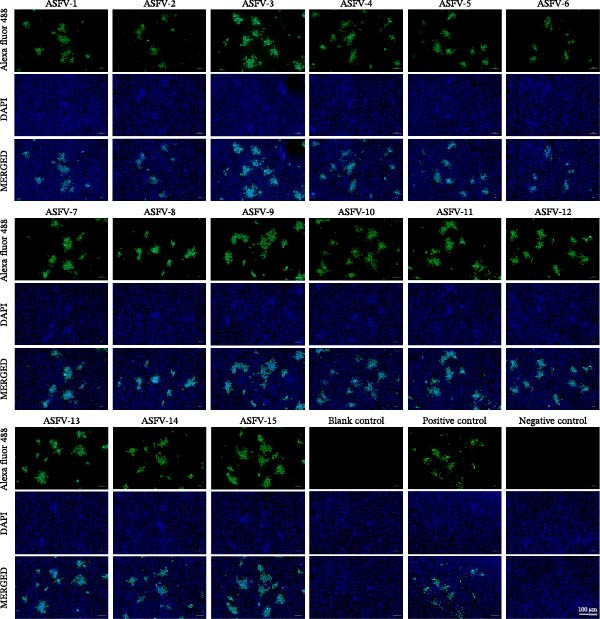
Evaluation of mAb specificity by IFA. IFA was performed to test the specificity of 15 anti‐ASFV mAbs. BK cells were infected with the ASFV SD strain, fixed, and probed with the respective mAbs. Binding was visualized with an Alexa Fluor 488‐labeled goat antimouse IgG secondary antibody (green fluorescence), and nuclei were counterstained with DAPI (blue). All mAbs exhibited specific fluorescent staining. Control wells contained PBS (blank), immunized mouse serum (positive control), or SPF mouse serum (negative control).

**Figure 3:**
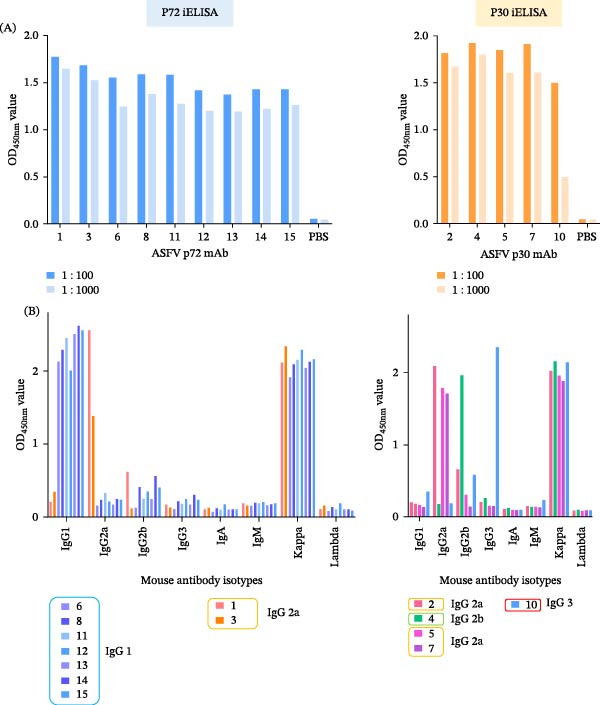
Characterization of mAb specificity and subclass. (A) Identification of target antigens by protein‐specific iELISA. Nine mAbs were specific for the major capsid protein p72, and five mAbs were specific for the phosphoprotein p30. Assays were performed at 1:100 and 1:1000 mAb dilutions; specific binding is indicated by optical density (OD) values as shown. These data are from a single experiment and were used for preliminary antibody identification. (B) Determination of antibody heavy‐chain isotype and light‐chain type. Data are from a single experiment for antibody characterization.

**Figure 4:**
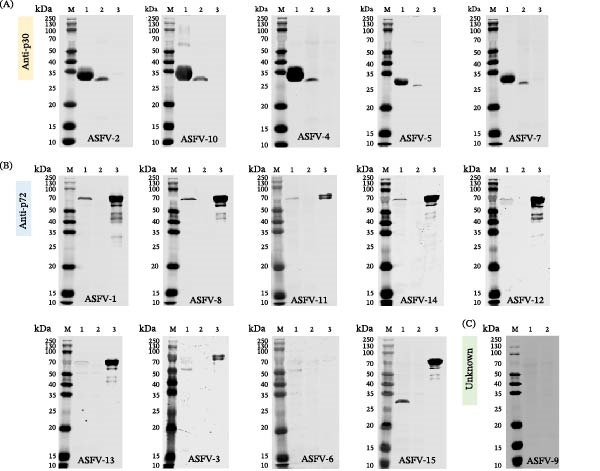
Western blot analysis of mAbs grouped by target specificity. (A) Anti‐p30 mAbs (ASFV‐2, 4, 5, 7, 10). Lane 1: purified recombinant p30 protein; Lane 2: ASFV‐infected BK2258 cell lysate; Lane 3: mock‐uninfected BK2258 cell lysate. (B) Anti‐p72 mAbs (ASFV‐1, 3, 6, 8, 11, 12, 13, 14, 15). Lane 1: ASFV‐infected BK2258 cell lysate; Lane 2: mock‐uninfected BK2258 cell lysate; Lane 3: purified recombinant p72 protein. (C) Undetermined specificity mAb (ASFV‐9). Lanes 1: ASFV‐infected BK2258 cell lysate; Lane 2: mock‐uninfected BK2258 cell lysate. Most p72‐specific mAbs recognized bands in both the infected lysate and purified p72. One mAb reacted only with the infected lysate, and one recognized ~72 kDa band in purified p72, but ~30 kDa band in the infected lysate. All p30‐specific mAbs detected ~30 kDa band in the corresponding positive samples. ASFV‐9 mAb showed no specific reactivity.

Following immunization of BALB/c mice with whole ASFV, splenocytes were fused with SP2/0 myeloma cells and 15 hybridomas were generated via limiting dilution. All mAbs demonstrated specific reactivity in IFA against ASFV‐infected cells (Figure 2). Western blot analysis confirmed that 14 mAbs specifically recognized proteins in ASFV‐infected cell lysates, while one (ASFV‐9) did not yield a clear band (Figure S1). Using a combination of mass spectrometry (on ASFV‐1 and ASFV‐2) and iELISA against 12 purified proteins (A104R, B407L, B646L, CP312R, A137R, K145R, KP177R, K205R, CP204L, D117L, CP123L, and B602L), the targets of 14 mAbs were identified: nine against the p72 (B646L) protein, five against p30 (CP204L), and one (ASFV‐9) against an unknown target (Figure 3A). Isotyping revealed all mAbs possessed kappa light chains, with heavy chains of IgG1 (7 mAbs), IgG2a (5 mAbs), IgG2b (1 mAb), and IgG3 (1 mAb) (Figure 3B). A follow‐up Western blot analysis showed that 7 of the 9 anti‐p72 mAbs recognized bands in both infected cell lysates and purified p72. Among the remaining anti‐p72 mAbs, one reacted only with infected cells and one detected divergent molecular weights (~72 kDa in purified p72 vs. ~30 kDa in lysates). All five anti‐p30 mAbs detected consistent ~ 30 kDa bands in both samples, while ASFV‐9 again showed no specific reactivity (Figure 4).

### 3.3. Establishment of the p72‐Based Immunochromatographic Test Strip

To identify mAb pairs for antigen detection, we screened our newly generated whole‐virus anti‐p72 mAbs alongside a preexisting panel of mAbs prepared against the p72 trimeric protein. Initial validation (Table 1) confirmed that all mAbs were detectable by a goat antimouse secondary antibody. In antigen‐specific testing, all mAbs except ASFV‐6 reacted strongly with the p72 trimer. The lack of reactivity from ASFV‐6 suggests that it is not p72‐specific; subsequently, it was excluded from further pairing tests.

**Table 1:** Analysis of monoclonal antibody reactivity.

Coated protein	Goat antimouse IgG	P72	Coated protein	Goat antimouse IgG
Gold‐labeled antibody			Gold‐labeled antibody	
ASFV‐1	+++	+++	P72‐2	+++
ASFV‐3	+++	+++	P72‐3	+++
ASFV‐6	+++	−	P72‐4	+++
ASFV‐8	+++	++	P72‐5	+++
ASFV‐11	+++	++	P72‐6	+++
ASFV‐12	+++	++	P72‐7	+++
ASFV‐13	+++	++	P72‐8	+++
ASFV‐14	+++	++	P72‐9	+++
ASFV‐15	+++	++	P72‐10	+++
P72‐1	+++	\	P72‐11	+++

*Note:* Test results were interpreted visually based on the presence and intensity of the red band at the test line. −, negative, no band; +, weak positive; ++, moderate positive; +++, strong positive. The symbol \ denotes that the test was not performed for the respective sample.

Cross‐pairing analysis of the mAbs (Tables 2 and 3) identified multiple antibody combinations exhibiting strong binding signals. The most effective pairings were observed between P72‐3 and P72‐6; P72‐6 and P72‐3/P72‐2; P72‐6 and ASFV‐3; and P72‐3 and ASFV‐1/ASFV‐11. These were followed by combinations involving ASFV‐3 and P72‐1/P72‐6/P72‐11/ASFV‐11; ASFV‐11 and ASFV‐1/P72‐3/P72‐2/P72‐11; and ASFV‐1 and P72‐1/P72‐2/P72‐3/P72‐6/P72‐11/ASFV‐3, all of which demonstrated favorable pairing performance. Among the experimental batches, the most consistent and effective pairing was ASFV‐3 as the detection antibody and P72‐6 as the capture antibody. The results showed that the combination of ASFV‐3 and P72‐6 showed the clearest and darkest red bands at all test concentrations, and there was no nonspecific color development in the negative control.

**Table 2:** Summary of monoclonal antibody cross‐pairing test results.

Capture antibody	P72‐1	P72‐2	P72‐3	P72‐4	P72‐5	P72‐6	P72‐7	P72‐8	P72‐9	P72‐10	P72‐11
Gold‐labeled antibody											
P72‐1	\	++	++	+	+	+	−	−	−	−	−
P72‐2	++	\	+	+	+	++	+	+	+	−	+
P72‐3	++	+	\	+	+	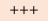	+	+	+	−	+
P72‐4	−	−	+	\	−	−	−	−	−	−	−
P72‐5	−	+	+	+	\	−	−	−	−	−	−
P72‐6	+	++	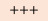	−	+	\	+	+	+	−	+
P72‐7	−	−	+	−	−	+	\	−	−	−	−
P72‐8	−	−	+	−	−	−	−	\	−	−	−
P72‐9	−	−	+	−	−	−	−	−	\	−	−
P72‐10	−	−	−	−	−	−	−	−	−	\	−
P72‐11	−	+	+	−	−	−	−	−	−	−	\
ASFV‐1	+	++	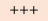	+	+	+	+	+	+	\	++
ASFV‐3	++	++	++	+	+	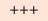	+	+	+	\	++
ASFV‐8	−	−	−	−	−	−	−	−	−	\	−
ASFV‐11	−	++	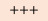	−	−	+	−	−	−	\	−
ASFV‐12	−	+	−	−	−	+	−	−	−	\	−
ASFV‐13	−	+	+	−	−	+	−	−	−	\	−
ASFV‐14	−	+	+	−	−	+	−	−	−	\	−
ASFV‐15	−	+	+	−	−	+	−	−	−	\	−

*Note*: Test results were interpreted visually based on the presence and intensity of the red band at the test line: − (negative, no band), + (weak positive), ++ (moderate positive), +++ (strong positive). The symbol \ denotes that the test was not performed for the respective sample.

**Table 3:** Summary of monoclonal antibody cross‐pairing test results.

Capture antibody	ASFV‐1	ASFV‐3	ASFV‐8	ASFV‐11	ASFV‐12	ASFV‐13	ASFV‐14	ASFV‐15
Gold‐labeled antibody								
P72‐1	++	++	−	−	\	\	\	\
P72‐2	++	+	−	++	−	−	−	−
P72‐3	++	+	−	++	−	+	+	+
P72‐4	+	+	−	−	\	\	\	\
P72‐5	+	+	−	−	\	\	\	\
P72‐6	+	++	−	+	+	+	+	+
P72‐7	−	+	−	−	\	\	\	\
P72‐8	−	−	−	−	\	\	\	\
P72‐9	+	+	−	−	\	\	\	\
P72‐10	+	+	−	−	\	\	\	\
P72‐11	++	++	−	++	+	+	+	+
ASFV‐1	\	++	−	++	+	+	+	+
ASFV‐3	++	\	−	−	+	+	+	+
ASFV‐8	−	+	\	−	\	\	\	\
ASFV‐11	+	++	−	\	+	+	−	−
ASFV‐12	+	+	−	−	\	\	\	\
ASFV‐13	+	+	−	−	\	\	\	\
ASFV‐14	−	+	−	−	\	\	\	\
ASFV‐15	−	+	−	−	\	\	\	\

*Note:* Test results were interpreted visually based on the presence and intensity of the red band at the test line. −, negative, no band; +, weak positive; ++, moderate positive; +++, strong positive. The symbol \ denotes that the test was not performed for the respective sample.

**Figure 5:**
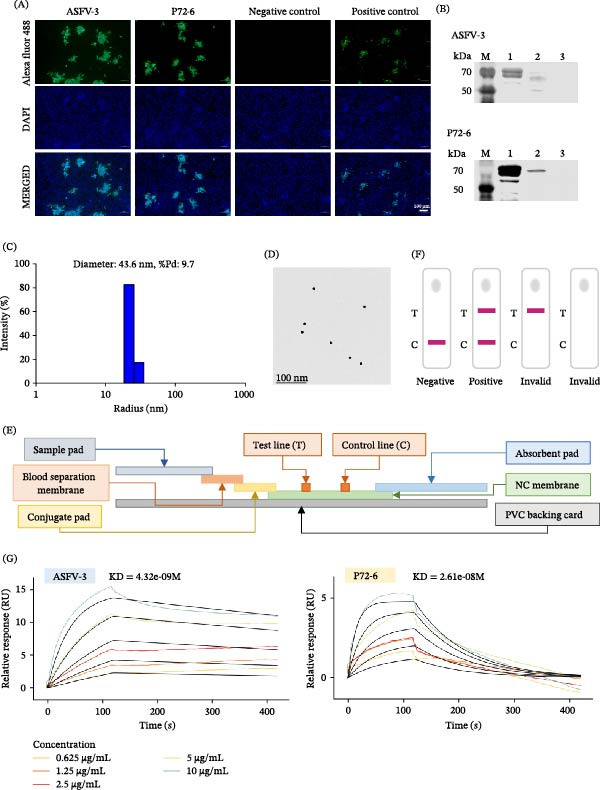
Characterization of candidate mAbs and design of an ASFV antigen detection strip. (A) IFA confirming the reactivity of mAbs ASFV‐3 and P72‐6 with ASFV‐infected BK2258 cells. Anti‐ASFV immune serum and SPF mouse serum served as positive and negative controls, respectively. (B) Western blot analysis of mAbs ASFV‐3 and P72‐6. Lane 1: purified p72 protein; Lane 2: ASFV‐infected BK2258 cell lysate; Lane 3: mock‐uninfected BK2258 cell lysate. (C) DLS size distribution of colloidal gold nanoparticles. (D) TEM of colloidal gold particles. (E) Schematic diagram of the lateral flow immunochromatographic strip. (F) Diagram illustrating result interpretation for the strip test (positive, negative, invalid). (G) SPR sensorgrams of ASFV‐3 and P72‐6 binding to recombinant p72 protein. Antibodies immobilized on CM5 chip (Biacore 8 K). p72 protein: 10−0.625 μg/mL (five concentrations) in PBS, 30 μL/min. Colored: raw data; black: 1:1 Langmuir fit. Association/dissociation: 120/300 s. The information regarding the kinetic parameters is shown in Table S3.

P72‐6 was derived from purified p72 immunization, and ASFV‐3 was identified from the inactivated ASFV immunization, both of which were confirmed to be p72‐specific by IFA and Western blot (Figure 5A,B). These antibodies were used to construct a lateral flow immunochromatographic test strip. P72‐6 was dispensed onto a NC membrane as the T line. Additionally, the size distribution of the colloidal gold nanoparticles was characterized using DLS. The analysis revealed a mean hydrodynamic diameter of 43.6 nm with a PDI of 0.097 (Figure 5C). TEM observation showed that the colloidal gold particles were spherical and uniformly dispersed (Figure 5D). These results indicate that the prepared colloidal gold nanoparticles exhibit high homogeneity and are suitable for antibody conjugation and test strip assembly. ASFV‐3 was conjugated to the 40 nm colloidal gold nanoparticles to serve as the detection conjugate. The conjugation efficiency was determined by BCA assay, giving an average efficiency of 90.8% ± 1.2% (*n* = 3) (Table S2), indicating stable and efficient antibody immobilization. Key manufacturing parameters were optimized to pH 7.5 for labeling, 20 µg/mL antibody for gold conjugation, 1.5 mg/mL for the T line coating, and 2 mg/mL for the C line (Table S1). The strip components sample pad, plasma separation membrane, conjugate pad, nitrocellulose membrane, and absorbent pad were assembled on a PVC backing (Figure 5E). A valid test requires a visible C line (composed of antispecies antibody). A positive result is defined by the appearance of both the T line and the C line, while a negative result shows only the C line. The absence of a C line indicates an invalid test (Figure 5F).

### 3.4. SPR Affinity Assay

The binding affinity of five antibodies with good pairing effect on the recombinant p72 protein was determined by SPR technology. The equilibrium dissociation constant, KD, of ASFV‐3 and P72‐6 was calculated to be 4.32e‐09M and 2.61e‐08M (Figure 5G). By using the 1:1 Langmuir model, both of which had high affinity at the nanomolar level, and the affinity of ASFV‐3 was about 6 times that of P72‐6. The KDs of the remaining three antibodies were 1.86e‐08M (ASFV‐1), 3.46e‐08M (ASFV‐11), and 1.94e‐08M (P72‐3) (Figure S2). ASFV‐3 had the highest affinity (4.32 nM), followed by P72‐6, ASFV‐1, and P72‐3 (about 19–26 nM), and ASFV‐11 was relatively low (34.6 nM). The kinetic parameters of all antibodies are summarized in Table S3.

### 3.5. Specificity Assessment

**Figure 6:**
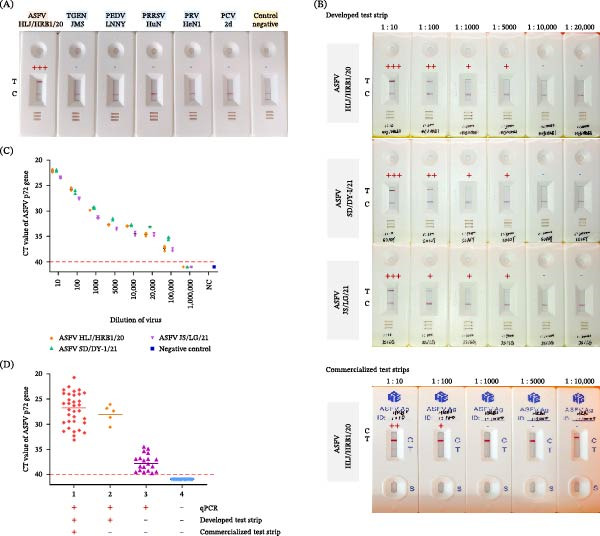
Analytical and clinical performance assessment of the developed colloidal gold test strip. (A) Specificity of the colloidal gold test strips. (B) Analytical sensitivity of the colloidal gold test strips. (C) Analytical sensitivity of the qPCR. Data from three independent replicates, error bars represent standard deviations. (D) Evaluating clinical sample detection. Groups are defined based on agreement between qPCR, the developed strip, and the commercial strip: Group 1: all positive; Group 2: qPCR and developed strip positive, commercial strip negative; Group 3: only qPCR positive; Group 4: all negative.

The specificity of the test strip was evaluated against a panel of common swine pathogens, including PCV‐2d, PRRSV HuN4, PRV HeN1, PEDV LN‐NY, and TGEV JMS strains. As shown in Figure 6A, only ASFV samples produced both the T line and the C line. Samples from all other pathogens produced only the C line. These results demonstrate that the test strip exhibits high specificity for ASFV, with no observed cross‐reactivity against the other pathogens tested.

### 3.6. Sensitivity Assessment

To determine the limit of detection (LOD), three independent replicate tests were performed on viral stocks from three ASFV strains (HLJ/HRB1/20, SD/DY‐Ⅰ/21, and JS/LG/21), which were subjected to serial tenfold dilution and tested with the lateral flow strip. In all three replicates, the detection limits for the three ASFV strains were 10^2.5^, 10^2.42^, and 10^2.30^ TCID_50_ per reaction(representative strip images are shown in Figure 6B; detailed results are provided in Table S4). Therefore, the LOD of the developed strip was defined as a 1:5000 dilution (~10^2.50^ TCID_50_ per reaction). In contrast, the commercial test strip exhibited a higher (less sensitive) limit of detection of 10^4.20^ TCID_50_ per reaction for the HLJ/HRB1/20 strain, demonstrating the superior sensitivity of our newly developed strip. The corresponding qPCR results at these dilutions, as shown in Figure 6C, were all positive, with CT values of 32.70, 31.63, and 33.47.

### 3.7. Reproducibility Test

As shown in Table 4, the test strips yielded positive results for all five qPCR‐positive samples across all replicates and batches and produced negative results for all five qPCR‐negative samples. The results were consistent across three replicates within the same batch and between different batches. The findings indicate that the newly established test strip exhibits excellent intrabatch and interbatch reproducibility.

**Table 4:** Intrabatch and interbatch reproducibility.

Batch	P1	P2	P3	P4	P5	N1	N2	N3	N4	N5
1	3/3	3/3	3/3	3/3	3/3	0/3	0/3	0/3	0/3	0/3
2	3/3	3/3	3/3	3/3	3/3	0/3	0/3	0/3	0/3	0/3
3	3/3	3/3	3/3	3/3	3/3	0/3	0/3	0/3	0/3	0/3

*Note:* P1–P5, qPCR‐positive samples; N1–N5, qPCR‐negative samples. Each sample was tested three times per batch. Results are expressed as number of positive results out of total replicates.

### 3.8. Clinical Sample Testing

A total of 90 samples (84 from infected pigs and 6 from healthy pigs) were tested in parallel using the new test strip, qPCR, and a commercial test strip. Compared to the commercial strip, the new test strip demonstrated an overall agreement of 93.33% (Table 5). Analysis of the qPCR‐positive samples revealed that the new test strip detected six samples earlier than the commercial comparator, with no observed false positives (Figure 6D). Furthermore, a trend toward earlier detection was noted: one positive anal swab was detected on 5 dpi by the new test strip, whereas most other positives for both strips were identified on 7 dpi (Table 6).

**Table 5:** Summary of compliance rate test results.

Assay	Commercialized test strips		
	Positive	Negative	Total
Developed test strip			
Positive	46^a^	6^c^	52
Negative	0^b^	38^d^	38
Total	46	44	90
Concordance rate	100.00%^e^	88.36%^f^	93.33%^g^

^a^The number of samples with positive results in both commercialized strips test and developed strip test.

^b^The number of samples with commercialized strips test results being positive and developed strip test results being negative.

^c^The number of samples with commercialized strips test result being negative, and developed strip test results being positive.

^d^The number of samples with negative results in both commercialized strips test and developed strip test.

^e^Sensitivity % = a/(a + b) × 100.

^f^Specificity % = d/(c + d) × 100.

^g^Coincidence rate % = (a + d)/(a + b + c + d) × 100.

**Table 6:** Comparison of detection results from oral and anal swabs in clinical samples.

Day postinfection	Sample type		1	2	3	4	5	6	7	8
3	Oral swab	qPCR	37.09	39.52	37.92	38.39	−	37.25	39.20	34.48
		Developed test strip	−	−	−	−	−	−	−	−
		Commercialized test strips	−	−	−	−	−	−	−	−
	Anal swab	qPCR	−	−	39.80	−	−	−	34.91	−
		Developed test strip	−	−	−	−	−	−	−	−
		Commercialized test strips	−	−	−	−	−	−	−	−
5	Oral swab	qPCR	35.77	38.43	39.24	36.80	39.66	−	−	38.49
		Developed test strip	−	−	−	−	−	−	−	−
		Commercialized test strips	−	−	−	−	−	−	−	−
	Anal swab	qPCR	36.96	−	39.60	−	−	36.97	−	32.98
		Developed test strip	−	−	−	−	−	−	−	+
		Commercialized test strips	−	−	−	−	−	−	−	−
7	Oral swab	qPCR	26.83	26.11	35.34	32.29	25.15	28.66	27.39	28.07
		Developed test strip	+	+	−	+	+	+	+	+
		Commercialized test strips	−	−	−	+	+	−	+	+
	Anal swab	qPCR	23.16	31.47	29.70	30.77	33.13	28.94	30.57	31.69
		Developed test strip	+	+	+	+	+	+	+	+
		Commercialized test strips	+	+	+	+	+	+	−	+
9	Oral swab	qPCR	/	24.17	25.93	27.42	23.79	23.97	29.42	/
		Developed test strip	/	+	+	+	+	+	+	/
		Commercialized test strips	/	+	+	+	+	+	+	/
	Anal swab	qPCR	/	28.50	29.65	29.42	25.55	27.11	26.09	/
		Developed test strip	/	+	+	+	+	+	+	/
		Commercialized test strips	/	+	+	+	+	+	+	/

*Note:* qPCR results are reported as Ct values (positive: Ct ≤ 40, shown as numerical values; negative: Ct > 40 or no signal, shown as “−”). Both test strips report “+” for positive and “–” for negative. “/” indicates no sample available.

## 4. Discussion

Based on the analysis of antigen recognition characteristics and methodological principles, mAbs obtained by whole‐virus particle screening may be more suitable as target antibodies for colloidal gold test strips. Although mAbs screened for a single protein are highly specific, the epitopes they recognize may be obscured by other structures in intact virus particles or differ from the native conformation, thus affecting their detection sensitivity for intact viruses. The mAb based on whole‐virus screening recognizes epitopes located on the natural surface of the virus and can efficiently bind to intact viral particles, which not only ensures high specificity but also significantly improves the affinity and sensitivity of the detection. Through pairing experiments, the antibody pairs with the strongest signal against natural viruses were screened, among which the coated antibodies were selected with high affinity and strong stability to firmly capture the virus‐gold label complex. Gold‐standard antibodies are selected with high labeling efficiency and fast reaction kinetics to ensure rapid binding and migration during chromatography [20, 27]. Based on this, we finally established the optimal antibody function combination, which not only revealed the reason why the sensitivity of a single recombinant protein antibody is limited in detecting intact virus, that is, it is more suitable as a static “trapper” rather than a dynamic “catching hand,” but also provides an efficient antibody pairing strategy for rapid detection technology based on intact pathogens: obtaining “capture antibodies” through natural pathogen immunity and combining highly specific recombinant protein antibodies as “trap antibodies.” It can achieve a synergistic improvement in detection sensitivity and specificity. As an important capsid protein with high conservation and strong immunogenicity in ASFV, p72 has always been a key target for diagnosis and vaccine development [14, 15, 28, 29]. Although a variety of p72‐based diagnostic methods have been established, the development of more accurate, reliable, and suitable rapid detection technologies suitable for grassroots sites is still an urgent need for current prevention and control work. Therefore, in this study, a colloidal gold immunochromatographic detection method based on ASFV p72 protein mAb was successfully established based on the advantages of whole‐virus immunity and p72 targeting specificity.

The 14 mAbs targeting p72 and p30 proteins obtained in this study cover all four isoforms of mouse IgG (IgG1, IgG2a, IgG2b, and IgG3) [30], and the light chains are all κ‐type, which is of great methodological and application significance. From an immunological perspective, whole‐virus immunity successfully stimulated a broad humoral immune response against ASFV‐dominant antigens, and the diversity of subtypes reflected the full activation of the immune system, laying the foundation for screening high‐affinity antibodies. At the diagnostic application level, different isotypes of antibodies have their own characteristics, as reported elsewhere. For instance, IgG1/IgG2b are generally considered to have higher stability [30, 31], while IgG2 a/IgG3 are thought to exhibit faster binding kinetics [32–34]. In the final pair screening, we found that the combination of one IgG2a antibody as the gold standard antibody and one IgG1 antibody as the coated antibody showed the best detection sensitivity. An efficient immunochromatography system can be designed by leveraging the distinct properties of antibody isoforms. For optimal performance, highly mobile antibodies with rapid kinetics (e.g., IgG2a from whole viral immune sources) can serve as the liquid‐phase “capture” element, while highly stable and specific recombinant antibodies (such as IgG1) can function as the immobilized “detection” element on the solid phase. However, the core of determining its ultimate performance remains the specificity, spatial accessibility, and conformational complementarity between pairs of each antibody’s recognition epitope rather than simple subtype classification. Of note, a unique monoclonal antibody, ASFV‐15, was obtained in this study. The antibody showed a strong reaction to the p72 protein in both ELISA and Western blot assays. In contrast, it tested negative for the p30 protein by ELISA but produced a strongly positive result in the Western blot, revealing a specific band at 30 kDa in the virus‐infected cell lysate but not at 72 kDa (Figure S3). This suggests that the mAb ASFV‐15 may recognize a linear epitope present on both p30 and p72. However, this epitope may be exposed only under denaturing conditions in p30 (hence the negative ELISA result), whereas it remains exposed in native p72. The lack of p72 bands in the viral lysate may be explained by rapid degradation or modification of p72 in infected cells, making it undetectable by Western blot [35, 36]. The unique performance of ASFV‐15 in recognizing the 30 kDa band in natural viral lysate and the 72 kDa band in recombinant p72 further confirms the unique advantage of whole‐virus immunization strategies in obtaining antibodies against natural conformational epitopes. Through intact viral particle immunization, antibodies that recognize the epitopes of antigens that have been exposed or processed during viral infection can be screened, which is of great value for the development of diagnostic reagents. In addition, the establishment of this antibody library not only provides key raw materials for the development of colloidal gold test strips but also lays an experimental foundation for the optimization of subsequent diagnostic methods, the expansion of different detection platforms, and in‐depth research on ASFV immunology.

To explain the high sensitivity of the test strips at the molecular level, we determined the affinity of the conjugated antibodies. In this study, the recombinant p72 protein was used to replace the intact ASFV virus particle for SPR affinity determination, mainly based on the following considerations: the large size of ASFV virus particles (about 260 nm), the risk of mass transport limitation and flow path blockage directly as an analyte, and the limited viral purification yield. The p72 protein is the main capsid protein of the virus, and the sequence is highly conserved, and the antibody against it can effectively identify the intact virus [13, 28]. Therefore, the affinity data of the p72 protein can reliably reflect the binding ability of the antibody to the virus. All fitted χ^2^ values were less than 0.5, indicating that the 1:1 Langmuir model was in good agreement with the experimental data. The affinity of ASFV‐3 is about six times that of P72‐6, which is highly consistent with the functional division of the two antibodies in the test strip: ASFV‐3, as a gold standard antibody (detection antibody), needs to quickly capture the viral antigen in the liquid phase during chromatography, and the higher affinity (especially the fast binding rate, ka = 1.69 × 10^5 M−1 s−1^) is conducive to improve the detection sensitivity and reaction speed. As a solid‐phase trapping antibody, P72‐6’s moderate affinity (kd = 1.11 × 10^−2 s−1^) is sufficient to stably bind to the captured complex while avoiding excessive nonspecific adsorption. Notably, the dissociation rate of ASFV‐3 (kd = 7.29 × 10^−4 s−1^) is much slower than that of P72‐6, indicating that the complex formed by ASFV‐3 with the antigen is more stable, which may be a key factor in maintaining signal intensity as a gold‐label antibody during long‐term chromatography. The antibodies used in this study were obtained by intact virus immunization, and the actual detection performance of the test strips against intact viruses has been verified (LOD of about 10^2.5^ TCID_50_ per reaction), so the SPR data and the performance of the test strips are in good agreement. In the future, nanoscale SPR technology or other methods should be used to further verify the direct binding of antibodies to intact virus particles.

In this study, the performance of the ASFV colloidal gold immunochromatography test strips was systematically evaluated. The results showed that the method had good detection ability for major domestic epidemic strains in China, and its minimum detection limit was 10^2.50^ TCID_50_ per reaction. Compared with the commercial test strips, the sensitivity of this test strip is about 50 times higher (the LOD of the commercial test strip is about 10^4.20^ TCID_50_ per reaction. In addition, this test strip can detect genotype Ⅰ, genotype Ⅱ, and genotype Ⅰ/Ⅱ. recombinant strains and has good genotype coverage. In the specific test, the test strip did not cross‐react with common porcine pathogens such as porcine circovirus type 2, porcine reproductive and respiratory syndrome virus, and porcine pseudorabies virus. The verification results of clinical samples showed that the test results of this method were in good agreement with the test results of commercial test strips, with a total concordance rate of 93.33% and clinical sensitivity and specificity of 100.00% and 88.36%, respectively. It is worth noting that in the dynamic monitoring of clinical samples, this test strip detected one positive sample from the anal swab on the 5th day after infection, while the commercial test strip began to detect positive on the 7th day, and this test strip can detect infection at least 2 days in advance, indicating that this test strip has the potential to detect antibodies earlier than the commercial kit, which is of great significance for early detection of infection and timely prevention and control measures. The above data show that the detection method established in this study has reliable detection performance and can meet the actual needs of on‐site rapid screening of ASF, especially suitable for grassroots scenarios such as farms, slaughterhouses, and border ports, which is conducive to early detection and early disposal and reduce the risk of epidemic transmission.

Although the colloidal gold immunochromatography method established in this study shows good field application performance in terms of sensitivity, specificity, and convenience, there are still certain limitations: although its detection sensitivity is better than that of the commercially available test strips, there is still a gap compared with laboratory qPCR methods, and missed detection may occur in samples with very early infection or very low viral load. And the long‐term stability needs to be further verified. In the future, portable readers can be developed to achieve semiquantitative detection, expand antibody pairs to higher sensitivity platforms such as fluorescence chromatography, and explore their application potential in therapeutic and vaccine evaluation [37–39].

The qPCR method recommended by WOAH targets the highly conserved B646L gene for ASFV nucleic acid detection. However, field monitoring data indicate that single‐site variations have occurred in the B646L gene of different virus strains [40]. To evaluate the conservation of the p72 protein as a diagnostic target and assess its potential mutation risk, we analyzed 170 ASFV reference sequences archived in the NCBI public database from January 2022 to April 2026. Focusing on China and neighboring countries, we selected 31 representative sequences (including three strains detected by the test strip) for multiple sequence alignment of the p72 protein (Figure S4 and Table S5). The alignment revealed three common mutation sites (S126A, V376I, and A383P) in nine p72 sequences from mainland China between 2022 and 2026. All three strains detected by the test strip carried these mutations, which is consistent with circulating strains in mainland China. An additional variant (R392H) was identified in only one isolate from Hong Kong. Compared with neighboring countries, the Indian sequence contained eight mutations, and the Philippine sequence had one variant, both of which were unique to their respective countries. These results indicate that the detection performance of the test strip against mainstream epidemic strains in mainland China is not affected by p72 mutations. However, it should be noted that this study did not perform fine epitope mapping of the monoclonal antibody used, nor did it experimentally test the Hong Kong strain carrying the R392H mutation; therefore, it remains unclear whether this mutation affects antibody recognition. The colloidal gold immunochromatographic assay developed in this study relies on the specific recognition of epitopes on the p72 protein by monoclonal antibodies. Amino acid variations at certain critical sites of p72 may affect antibody affinity, potentially leading to reduced sensitivity or false‐negative results. Therefore, with the ongoing prevalence and mutation of ASFVs in nature, continuous monitoring of p72 protein variations and regular evaluation of their impact on immunological diagnostic methods are essential to maintain diagnostic reliability.

In summary, a colloidal gold immunochromatography detection method based on ASFV p72 protein mAb was designed, which has good specificity, sensitivity, and accuracy compared with commercial colloidal gold detection test strips. In addition, the method is simple to operate and does not require complex sample preparation, making it suitable for on‐site rapid initial screening of ASFV.

## Author Contributions

**Wan Wang**: formal analysis, methodology, writing – original draft, writing – review and editing. **Weldu Tesfagaber**: formal analysis, methodology, writing – original draft, writing – review and editing. **Wenzhuang Zhu**: formal analysis, investigation, methodology, visualization. **Li Yin**: formal analysis, visualization, validation. **Fan Liu**: formal analysis, visualization, validation. **Runyi Yao**: formal analysis, visualization, validation. **Shangcong Wu**: formal analysis, visualization, validation. **Fang Li**: data curation, formal analysis, validation. **Encheng Sun**: data curation, formal analysis, validation. **Zhenjiang Zhang**: data curation, formal analysis, validation. **Zhigao Bu**: conceptualization, supervision. **Geng Meng**: conceptualization, supervision. **Yuanmao Zhu**: conceptualization, supervision, writing – review and editing. **Dongming Zhao**: conceptualization, supervision, writing – original draft, writing – review and editing.

## Funding

This work was funded by the National Science Foundation (Grant 32503011), China Postdoctoral Science Foundation (Grant 2024M763617), Innovation Program of Chinese Academy of Agricultural Sciences (Grant CAAS‐CSLPDCP‐202301), the Heilongjiang Provincial Natural Science Foundation of China (Grant JQ2023C005), and Central Public‐Interest Scientific Institution Basal Research Fund (Grant CAAS‐ZDRW202409).

## Disclosure

Each financially supporting body has no specific role in the conceptualization, design, data collection, analysis, decision to publish, or preparation of the manuscript.

## Ethics Statement

All procedures involving animals were carried out in strict accordance with the recommendation in the guide for the care and use of laboratory animals of the Ministry of Science and Technology of the People’s Republic of China. The protocols were approved by the Institutional Animal Care and Use Committee (IACUC) of the Harbin Veterinary Research Institute (HVRI), Chinese Academy of Agricultural Sciences (CAAS). The IACUC approval numbers are 210917‐01 and 221010‐02‐GR.

## Conflicts of Interest

The authors declare no conflicts of interest.

## Supporting Information

Additional supporting information can be found online in the Supporting Information section.

## Supporting information


**Supporting Information** Table S1: Optimization of CG‐ICS conditions. Table S2: BCA assay results for conjugation efficiency of ASFV‐3 to colloidal gold. Table S3: SPR affinity parameters of anti‐p72 mAbs. Table S4: Independent repeatability of the test strip for three ASFV strains at different dilutions. Table S5: List of 31 reference ASFV isolates for p72 alignment. Figure S1: Western blot analysis of anti‐ASFV mAbs. Cell lysates were probed with each mAb. Lane 1: ASFV‐infected BK2258 cell lysate; Lane 2: mock‐uninfected BK2258 cell lysate. Fourteen mAbs specifically recognized a band in the infected lysate (Lane 1) but not in the mock‐infected control (Lane 2). One mAb showed no specific reactivity. Figure S2: SPR sensorgrams of mAbs ASFV‐1, ASFV‐11, and P72‐3 binding to recombinant p72 protein. Colored: raw data; black: 1:1 Langmuir fit. The information regarding the kinetic parameters is shown in Supporting Table S3. Figure S3: Confirming the characterization of ASFV‐15 by ELISA and Western blot. (A) ELISA reactivity of ASFV‐15 with purified p72 and p30 proteins. Data are presented as mean OD450 ± SD (*n* = 3 independent experiments). ASFV‐15 showed strong binding to p72 at both 1:100 and 1:1000 dilutions, but no reactivity with p30 under native conditions. (B) Western blot analysis of ASFV‐15. Lane 1: ASFV‐infected BK2258 cell lysate; Lane 2: mock‐uninfected BK2258 cell lysate; Lane 3: purified p72 protein; Lane 4: purified p30 protein. ASFV‐15 detected a band around 30 kDa (expected size for p30), but not around 72 kDa (expected size for p72). Figure S4: Multiple sequence alignment of the p72 protein from 31 reference ASFV isolate. The alignment comprises three strains detected by our test strip (HLJ/HRB1/20, SD/DY‐Ⅰ/21, JS/LG/21; collected before 2022) and 28 isolates collected between 2022 and 2026. These sequences cover all 16 Chinese strains (7 of which are from Hong Kong) as well as 12 representative samples from neighboring countries (Vietnam, Russia, Singapore, Thailand, South Korea, India, Japan, the Philippines, and Sri Lanka). The conserved residues are marked in blue; the variable sites are highlighted in a white color. Detailed sequence information is provided in Table S5.

## Data Availability

The data that support the findings of this study are available from the corresponding author upon reasonable request.
