# Supplementary material for: A Rapid p72‐Targeting Colloidal Gold Immunochromatographic Strip for African Swine Fever Virus Detection
Source: Transbound Emerg Dis. 2026 Jun 5;2026:3198773. doi: 10.1155/tbed/3198773 (PMC13238504; doi:10.1155/tbed/3198773)
Supplement: Supplementary file 1 — Supporting Information Table S1: Optimization of CG‐ICS conditions. Table S2: BCA assay results for conjugation efficiency of ASFV‐3 to colloidal gold. Table S3: SPR affinity parameters of anti‐p72 mAbs. Table S4: Independent repeatability of the test strip for three ASFV strains at different dilutions. Table S5: List of 31 reference ASFV isolates for p72 alignment. Figure S1: Western blot analysis of anti‐ASFV mAbs. Cell lysates were probed with each mAb. Lane 1: ASFV‐infected BK2258 cell lysate; Lane 2: mock‐uninfected BK2258 cell lysate. Fourteen mAbs specifically recognized a band in the infected lysate (Lane 1) but not in the mock‐infected control (Lane 2). One mAb showed no specific reactivity. Figure S2: SPR sensorgrams of mAbs ASFV‐1, ASFV‐11, and P72‐3 binding to recombinant p72 protein. Colored: raw data; black: 1:1 Langmuir fit. The information regarding the kinetic parameters is shown in Supporting Table S3. Figure S3: Confirming the characterization of ASFV‐15 by ELISA and Western blot. (A) ELISA reactivity of ASFV‐15 with purified p72 and p30 proteins. Data are presented as mean OD450 ± SD (n = 3 independent experiments). ASFV‐15 showed strong binding to p72 at both 1:100 and 1:1000 dilutions, but no reactivity with p30 under native conditions. (B) Western blot analysis of ASFV‐15. Lane 1: ASFV‐infected BK2258 cell lysate; Lane 2: mock‐uninfected BK2258 cell lysate; Lane 3: purified p72 protein; Lane 4: purified p30 protein. ASFV‐15 detected a band around 30 kDa (expected size for p30), but not around 72 kDa (expected size for p72). Figure S4: Multiple sequence alignment of the p72 protein from 31 reference ASFV isolate. The alignment comprises three strains detected by our test strip (HLJ/HRB1/20, SD/DY‐Ⅰ/21, JS/LG/21; collected before 2022) and 28 isolates collected between 2022 and 2026. These sequences cover all 16 Chinese strains (7 of which are from Hong Kong) as well as 12 representative samples from neighboring countries (Vietnam, Russia, Singa [file TBED-2026-3198773-s001.docx]

**Supplementary Tables**

Table S1 Optimization of CG-ICS conditions

| Antibody | Coating concentration / Dispensing volume | 1:1000 | 1:2000 | 1:4000 | 1:5000 | 1:10000 | 1:15000 | Sample diluent |
| --- | --- | --- | --- | --- | --- | --- | --- | --- |
| P72-6 | 0.5 mg/ml | + | + | - | - | - | - | - |
|  | 1 mg/ml | + | + | + | - | - | - | - |
|  | 1.5 mg/ml | + | + | + | + | - | - | - |
|  | 2 mg/ml | + | + | + | + | + | + | + |
| Goat anti-mouse IgG | 0.5 mg/ml | + | + | + | + | + | \ | \ |
|  | 1 mg/ml | + | + | + | + | + | \ | \ |
|  | 2 mg/ml | ++ | ++ | ++ | ++ | ++ | \ | \ |
|  | 3 mg/ml | ++ | ++ | ++ | ++ | ++ | \ | \ |
| ASFV-3 | 6 μL/cm | + | + | + | - | - | - | - |
|  | 8 μL/cm | + | + | + | + | - | - | - |
|  | 10 μL/cm | + | + | + | + | + | + | + |

Note: The ASFV HLJ/HRB1/20 strain, with a titer of 10^7.20^ TCID_50_/mL. Test results were interpreted visually based on the presence and intensity of the red band at the test line: - (negative, no band), + (weak positive), ++ (moderate positive), +++ (strong positive). The symbol \ denotes that the test was not performed for the respective sample.

Table S2 BCA assay results for conjugation efficiency of ASFV-3 to colloidal gold

| Experimental Replicate | Total Antibody Concentration Before Conjugation (μg/mL) | Free Antibody Concentration After Conjugation (μg/mL) | Conjugation Efficiency (%) |
| --- | --- | --- | --- |
| 1 | 20 | 1.9 | 90.5 |
| 2 | 20 | 1.8 | 91 |
| 3 | 20 | 2 | 90 |
| Average ± SD (n=3) | 20.0 ± 0.0 | 1.9 ± 0.1 | 90.5 ± 0.5 |

Note: Data are presented as mean ± SD (n = 3)

Table S3 SPR affinity parameters of anti-p72 mAbs.

| Antibody | ka (M⁻¹s⁻¹) | kd (s⁻¹) | KD (M) | Rmax(RU) | Chi^2^ |
| --- | --- | --- | --- | --- | --- |
| ASFV-3 | 1.69e+05 | 7.29e-04 | 4.32e-09 | 15.08 | 0.47 |
| P72-6 | 4.25e+05 | 1.11e-02 | 2.61e-08 | 5.69 | 0.17 |
| ASFV-1 | 1.83e+05 | 3.41e-03 | 1.86e-08 | 9.81 | 0.44 |
| ASFV-11 | 9.98e+04 | 3.45e-03 | 3.46e-08 | 11.49 | 0.42 |
| P72-3 | 2.70e+05 | 5.23e-03 | 1.94e-08 | 3.94 | 0.11 |

Note: ka, association rate constant; kd, dissociation rate constant; KD, equilibrium dissociation constant; Rmax, maximum binding response; Chi^2^, chi-square value.

Table S4 Independent repeatability of the test strip for three ASFV strains at different dilutions.

| Virus strain | Replicate | 1:10 | 1:100 | 1:1000 | 1:5000 | 1:10000 | 1:20000 |
| --- | --- | --- | --- | --- | --- | --- | --- |
| HLJ/HRB1/20 | 1 | +++ | ++ | + | + | - | - |
|  | 2 | +++ | ++ | + | + | - | - |
|  | 3 | +++ | ++ | + | + | - | - |
| SD/DY-Ⅰ/21 | 1 | +++ | ++ | + | + | - | - |
|  | 2 | +++ | ++ | + | + | - | - |
|  | 3 | +++ | ++ | + | + | - | - |
| JS/LG/21 | 1 | +++ | ++ | + | + | - | - |
|  | 2 | +++ | ++ | + | + | - | - |
|  | 3 | +++ | ++ | + | + | - | - |

Note: Test results were interpreted visually based on the presence and intensity of the red band at the test line: - (negative, no band), + (weak positive), ++ (moderate positive), +++ (strong positive).

Table S5 List of 31 reference ASFV isolates for p72 alignment

| No. | GenBank accession No. | Isolate name | Country | Year | Genotype |
| --- | --- | --- | --- | --- | --- |
| HLJ/HRB1/20 | MW656282 | Pig/Heilongjiang/HRB1/2020 | China | 2020 | Ⅱ |
| SD/DY-Ⅰ/21 | MZ945537 | Pig/SD/DY-I/2021 | China | 2021 | Ⅰ |
| JS/LG/21 | OQ504956.1 | Pig/Jiangsu/LG/2021 | China | 2021 | Ⅰ/Ⅱ |
| 1 | OQ504954.1 | Pig/Henan/123014/2022 | China | 2022 | Ⅰ/Ⅱ |
| 2 | OQ504955.1 | Pig/Inner Mongolia/DQDM/2022 | China | 2022 | Ⅰ/Ⅱ |
| 3 | OR180113.1 | ASFV JS | China | 2022 | Ⅱ |
| 4 | OR290104.2 | CN/GD/2022 | China | 2022 | Ⅱ |
| 5 | PP478517.1 | NJS23-1 | China | 2023 | Ⅰ/Ⅱ |
| 6 | PP478518.1 | NJS23-2 | China | 2023 | Ⅰ/Ⅱ |
| 7 | PP712068.1 | JX23-01 | China | 2023 | Ⅰ/Ⅱ |
| 8 | PP712069.1 | JX23-02 | China | 2023 | Ⅰ/Ⅱ |
| 9 | PX667021.1 | ASFV_HN10005 | China | 2023 | Ⅰ/Ⅱ |
| 10 | PV400255.1 | ASFV/HKWB2022TP-00522 | China: Hong Kong | 2022 | Ⅱ |
| 11 | PV400256.1 | ASFV/HKWB2022S-10414 | China: Hong Kong | 2022 | Ⅱ |
| 12 | PV400257.1 | ASFV/HKWB2022SK-13869 | China: Hong Kong | 2022 | Ⅱ |
| 13 | PX277557.1 | HK/DP/2023/LFS-12807-34 | China: Hong Kong | 2023 | Ⅱ |
| 14 | PX277558.1 | HK/DP/2023/ST-14219-23/1 | China: Hong Kong | 2023 | Ⅱ |
| 15 | PX277559.1 | HK/DP/2024/KT-00117-2/16 | China: Hong Kong | 2024 | Ⅰ/Ⅱ |
| 16 | PX277560.1 | HK/DP/2024/ST-00662-2 | China: Hong Kong | 2024 | Ⅱ |
| 17 | OR290060.1 | ASF/IND/Mudumalai/CAD/685/22/W_PIG | India | 2022 | Ⅱ |
| 18 | LC862876.1 | ASFV/Japan/2023/3-1 | Japan | 2023 | Ⅰ/Ⅱ |
| 19 | LC862879.1 | ASFV/Japan/2023/4-1 | Japan | 2023 | Ⅰ/Ⅱ |
| 20 | PP737712.1 | NEC20230726003 | Philippines | 2023 | Ⅱ |
| 21 | PX454813.1 | BA25-0107 | Philippines | 2024 | Ⅱ |
| 22 | PP348677.1 | Primorsky 2023 DP-4560.Rec | Russia | 2023 | Ⅰ/Ⅱ |
| 23 | OR135685.1 | SG/NParks/A-MAM-2023-02-00021 | Singapore | 2023 | Ⅱ |
| 24 | PP320357.1 | ASFV-PJ-Vacln3 | South Korea | 2023 | Ⅱ |
| 25 | PV423385.1 | ASFV/SL/4/2024 | Sri Lanka | 2024 | Ⅱ |
| 26 | PX119974.1 | TH2_24/RB | Thailand | 2024 | Ⅱ |
| 27 | PQ010732.1 | rASF12-avac02 | VietNam | 2023 | Ⅰ/Ⅱ |
| 28 | PQ629526.1 | VN/sows_32005/23 | VietNam | 2023 | Ⅱ |

Note: The 31 sequences include three strains detected by our test strip (HLJ/HRB1/20, SD/DY-Ⅰ/21, JS/LG/21; collected before 2022) and 28 isolates collected between 2022 and 2026. Among the latter, 16 are from China (including 7 from Hong Kong) and 12 are representative isolates from neighboring countries (Vietnam, Russia, Singapore, Thailand, South Korea, India, Japan, the Philippines, and Sri Lanka).

**Supplementary Figures**


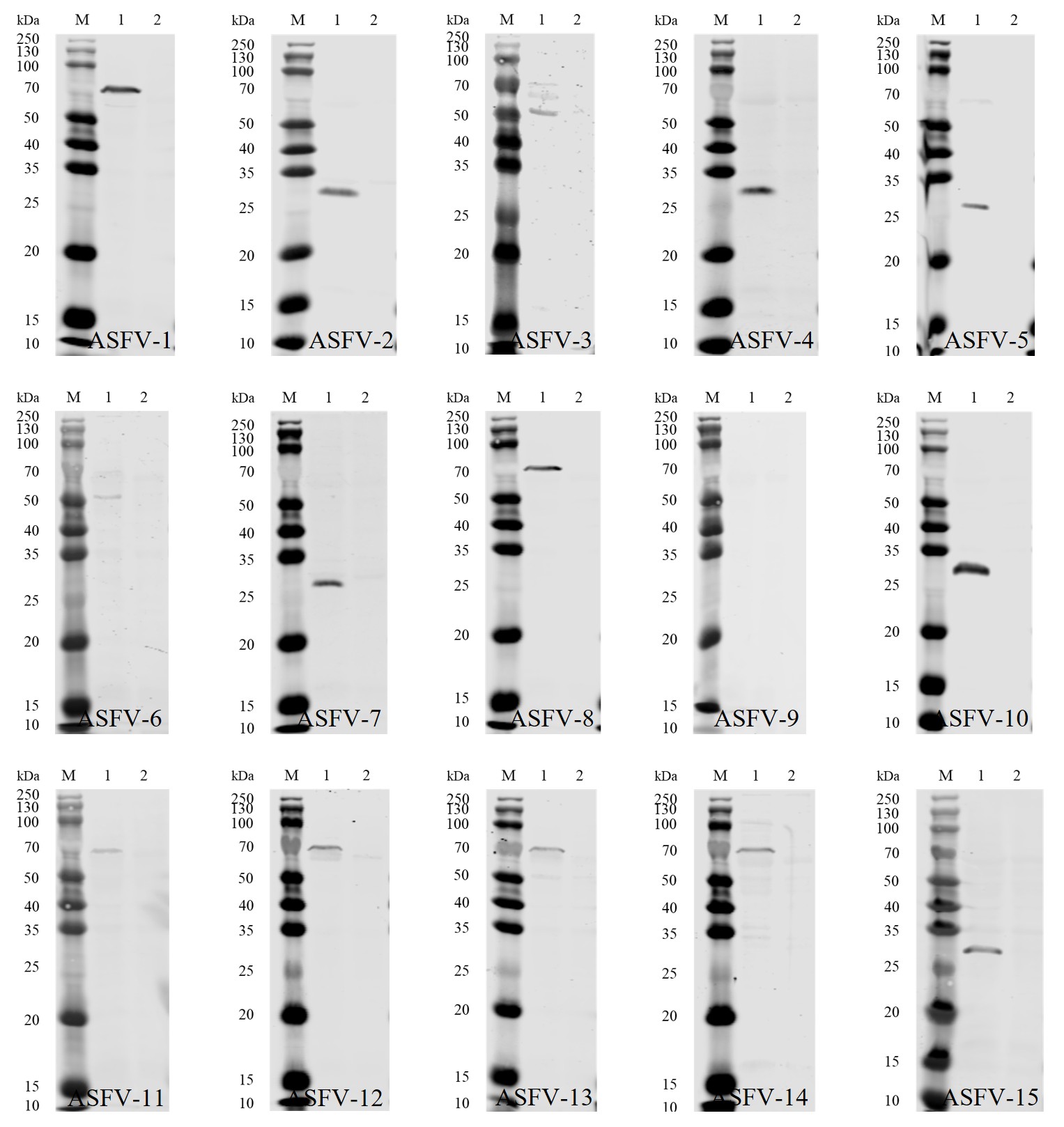


Figure S1. Western blot analysis of anti-ASFV mAbs


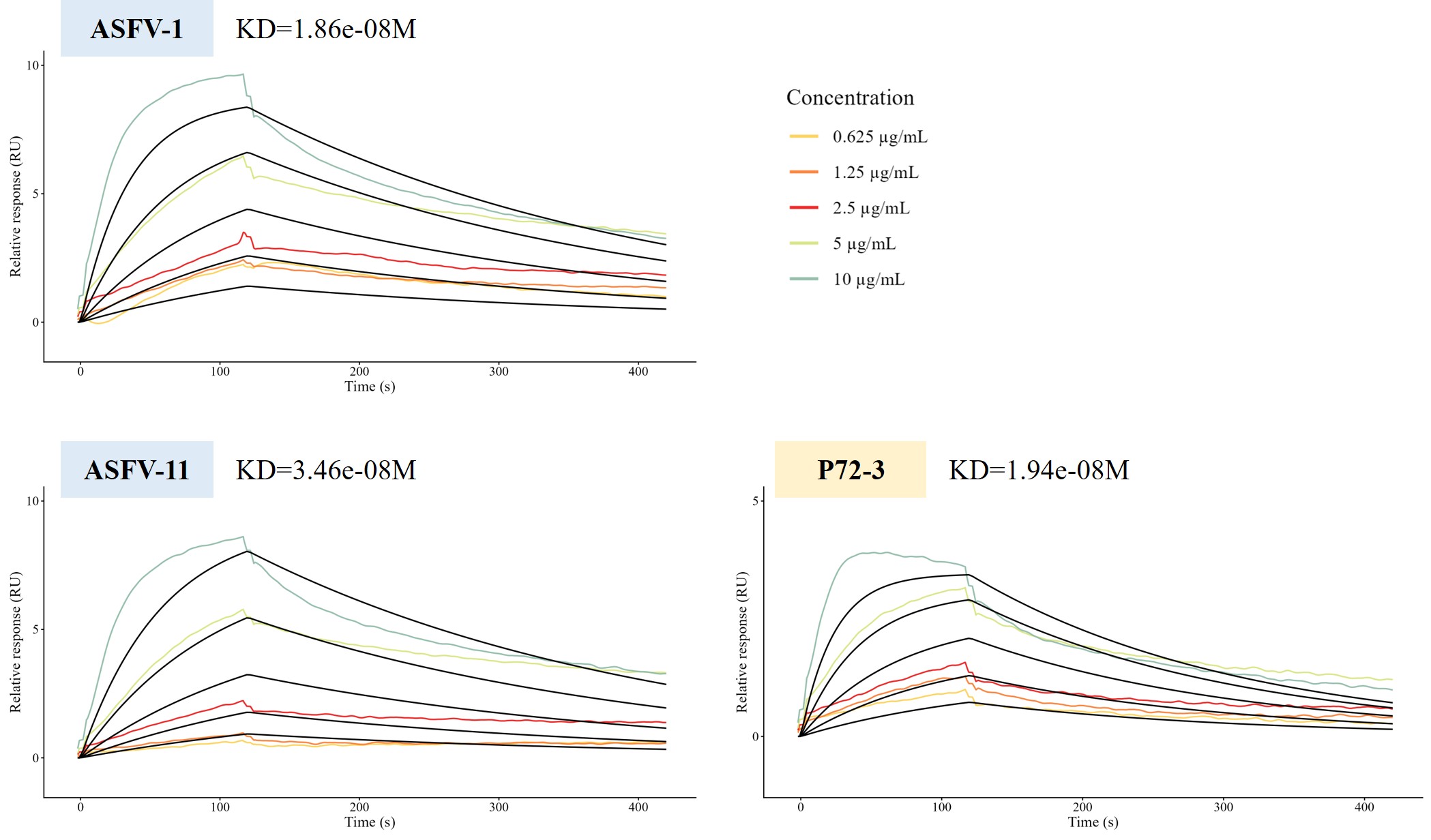


Figure S2. SPR sensorgrams of mAbs ASFV-1, ASFV-11, and P72-3 binding to recombinant p72 protein


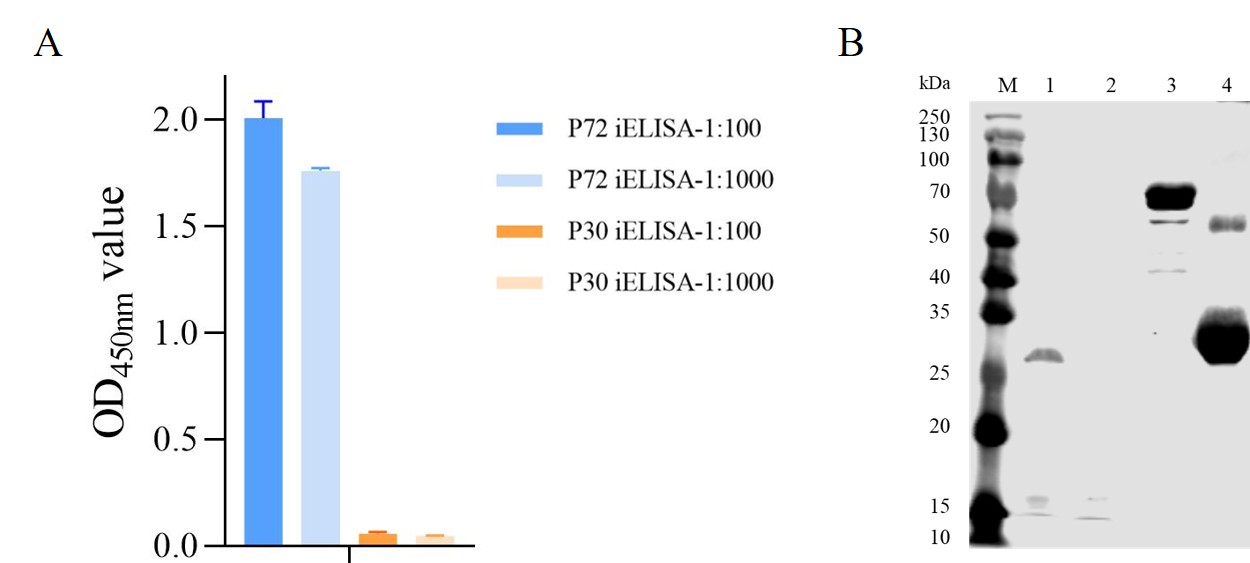


Figure S3. Confirming the characterization of ASFV-15 by ELISA and western blot


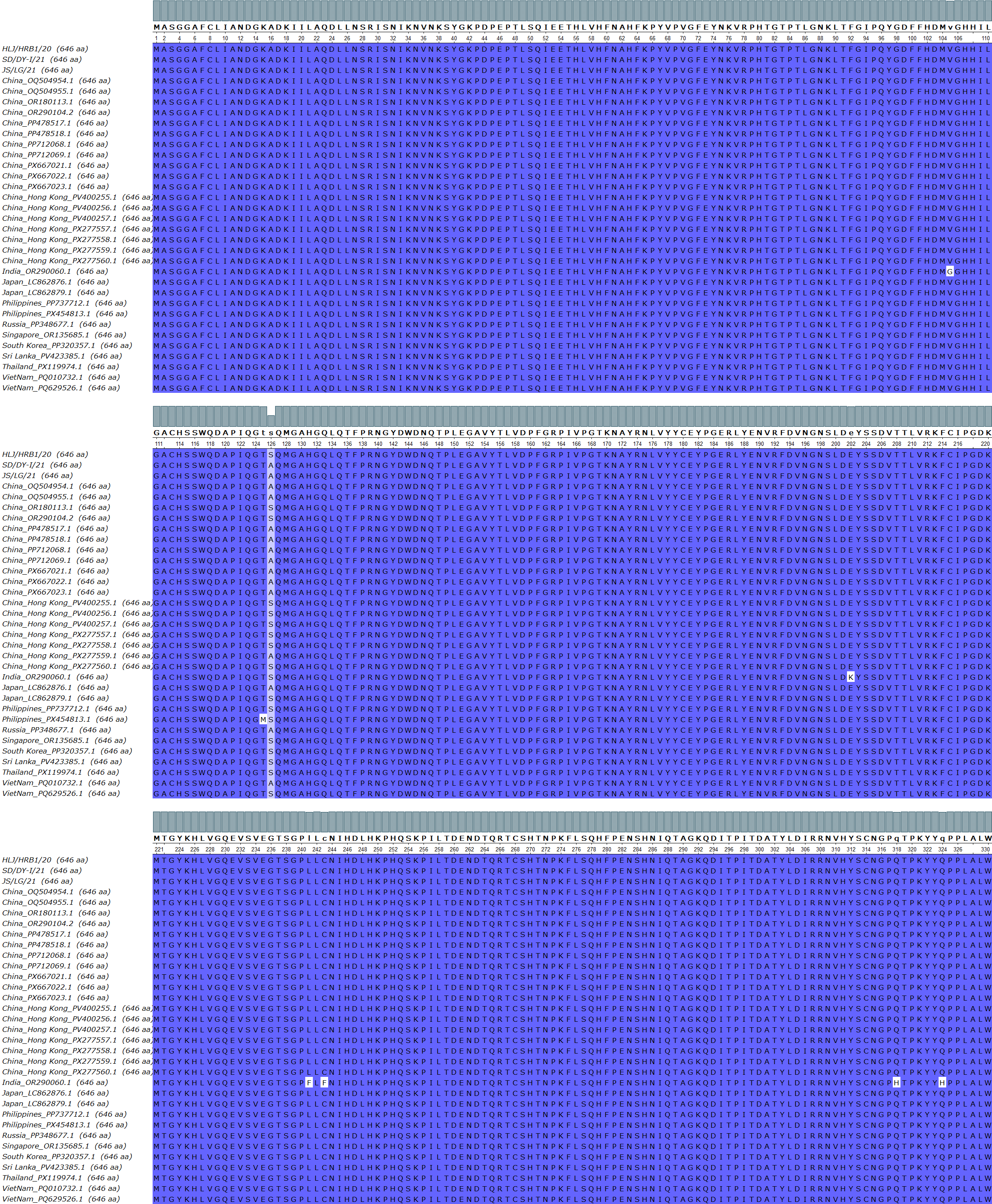


Figure S4. Multiple sequence alignment of the p72 protein from 31 reference ASFV isolates


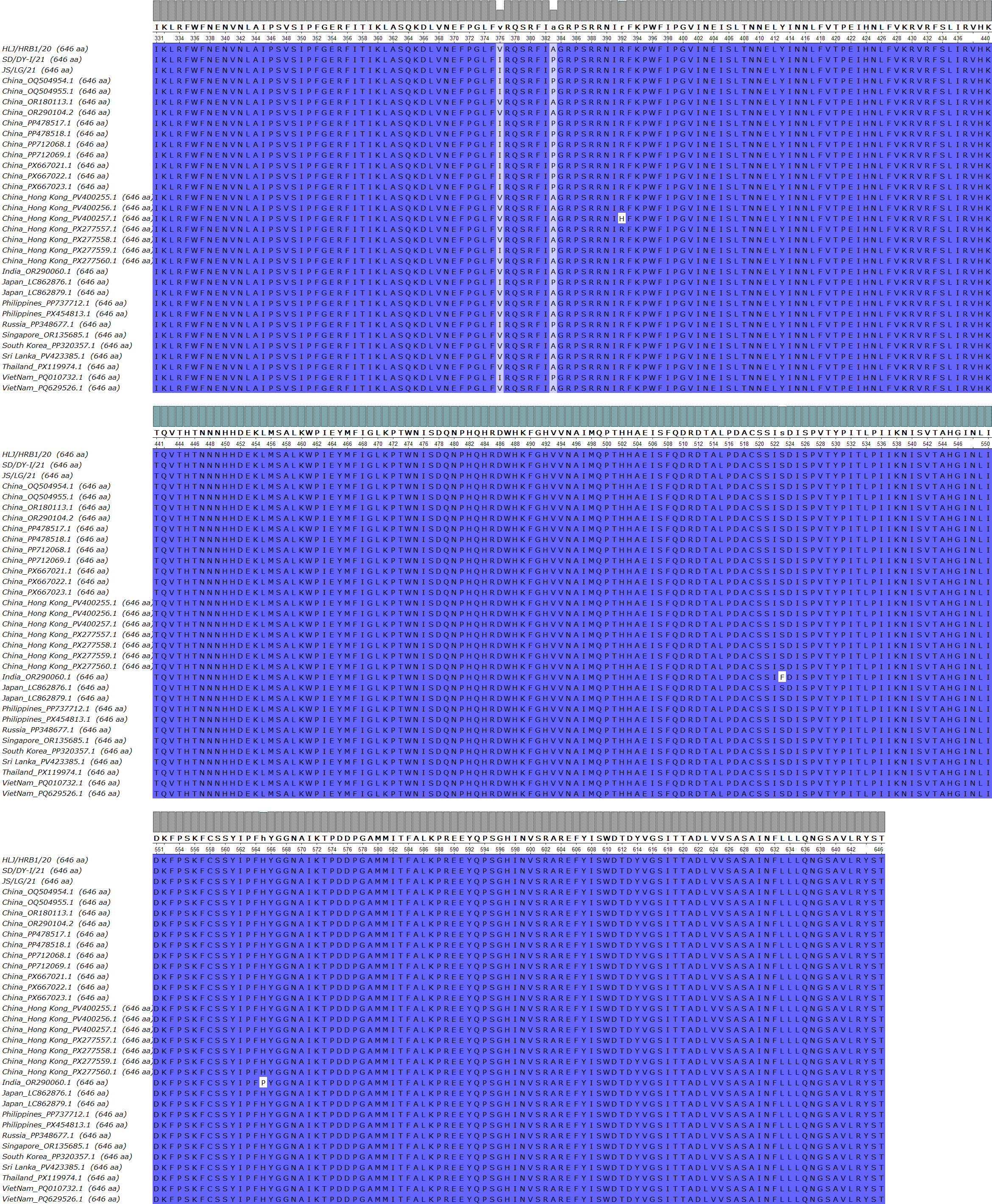


Figure S4 (continued)
